# Technology from traditional knowledge - *Vrikshayurveda*-based expert system for diagnosis and management of plant diseases

**DOI:** 10.1016/j.jaim.2023.100853

**Published:** 2024-01-13

**Authors:** Laxmi B. Rananavare, Sanjay Chitnis

**Affiliations:** a REVA University, Bangalore, India; b RV University, Bangalore, India

**Keywords:** Experta, Inference engine, Knowledge base, Kapha, Vata, Pitta

## Abstract

Vrikshayurveda (An ancient Indian science of plant life) includes complete plant-life knowledge compendium of plant physiology, horticulture, pathology, and treatment. Though translation of the manuscript is available, the knowledge contained in the translation is not easily accessible to ordinary farmers who want answers to their specific problems or researchers who want references for specific topics without having to read the complete book. This research work proposes to convert the knowledge in the manuscript form to an expert system form which can provide the solutions to specific queries from the farmers and agriculture stakeholders. A rule based expert system using backward chaining Expert System is developed. The database in this design has ten diseases. The evaluation is done for all the dataset. The results are compatible with the expert's diagnosis. Thus the users can get comprehensive information on Vriksha-Ayurvedic expertise on all elements of disease and plant protection.

## 1 Introduction

### 1.1 Background of Vrikshayurveda

Plants, like humans, experience joy and sorrow as a result of their existence. Chemical fertilizers and insecticides are now used to increase production. Indiscriminate usage leads to major environmental and health risks. It's intriguing to learn that ancient India possessed not only a human and animal medical science, but also the science of plant life. The term “Vrikshayurveda” has been in use by the time Kautilya (296–321 BC) compiled his “Arthasastra”. The next document on Vrikshayurveda, a very brief one, was included in “Brhat Samhita” by Varahamihira (505–581 AD). The two text compiled in the 11th century AD: Surapala's Vrikshayurveda (c. 1000) [1,2] and Vrikshayurveda chapter in Lokopakara composed by Chavundaraya (1025). In the 12th century AD, Chalukya King, Someshvardeva compiled an encyclopedia “Abhilashitarthachitamani”or “Manasollasa” in which a full chapter on Vrikshayurveda was included. In 13th-century AD text titled “Upavanavinoda”, which deals with landscape gardening. Upavanavinoda was compiled by Sarangadhara, a courtier and scholar in the court of King Hammira. In the court of great Maharana Pratap, a scholar, Chakrapani Mishra, compiled (c.1577 AD), adding his own experience, the text “Vishvavallabha”, which has contents similar to Surapala's Vrikshayurveda, with a good deal of additional information. Chronologically the last text available is “Shivatatvaratnakara” (in Kannada) compiled by King Basavaraja of Keladi, now in Karnataka; it has a chapter on “Vrikshayurveda” [3]. In general, Vrikshayurvedas deal with the following aspects: Detection of underground water; spacing between trees; methods of propagation; preparation of pits for planting; seed treatments; nourishment; protection; and some other relevant information. Vrikshayurveda, or “the science of plant life” focusses not only on curing plant diseases and protecting plants from insects and fungi, but also to keep them healthy in a sustainable way. Ayurveda's fundamental goal is to preserve or restore the right balance of the three doshas(defects): *Vata*, where the air and space components predominate; *Pitta*, where the fire element predominates; and *Kapha* (where the earth and water elements dominate. These ideas are also grafted onto the plants in Surapala's work. He asserts that the Ayurvedic lens should be used to view the plant condition, health-illness, causes, and treatments, among other things. Surapala promotes a comprehensive approach to crop management. He emphasizes the importance of using appropriate soils, good seeds and pre-sowing treatment of seeds, growing intercrops, having an optimal plant population, balanced nutrition, optimal water use, timely weeding, protection from disorders by using herbal products or dead animal wastes, harvesting at the appropriate stage, and drying and storing of seeds.

Plant Diseases: The diseases of all types of trees are stated to be of two varieties: internal and external. The internal ones are caused by vata, pitta and kapha; External ones are those which are caused by insects, cold weather, wind and sun (high temperature) etc. [4].

When a plant is diseased, the leaves turn yellow (etiolated), the buds do not form or their growth is halted, the branches turn dry, and sap leaks from the branches. The goal of *Vrikshayurveda* is to keep *Vata, Pitta,* and *Kapha* in good balance.

**Fig. 1:**
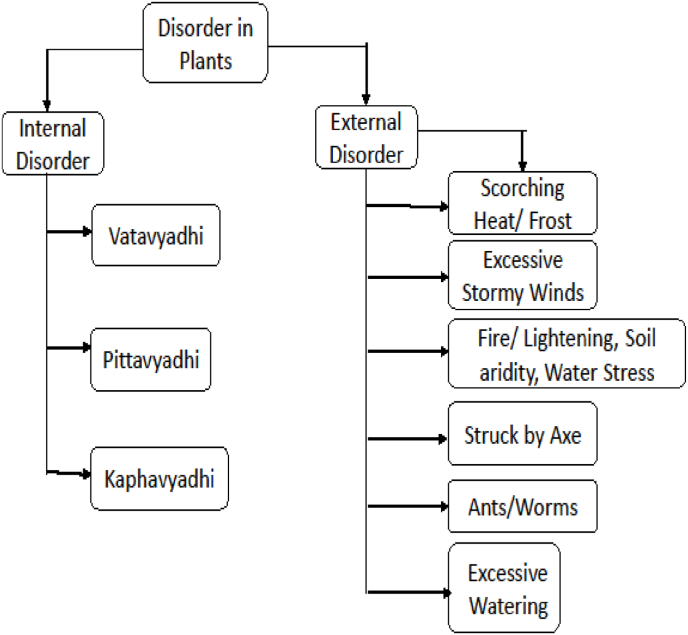
Classification of disorder in Plant.

The two types of disorder in plants are shown in Fig. 1. Here the Sanskrit term *vyadhi* stands for disease. The description of the diseases are shown in appendix Table 2.

### 1.2 Why task-oriented dialogue expert system?

The Expert systems incorporated with “Dialog” are capable of asking appropriate questions when necessary and collecting the answers to direct the line of reasoning and guide the conversation toward the correct solution. Besides, an explanation facility is provided to explain the reasoning behind its conclusion.

Unlike Chabot that aim to converse with humans in an open domain, task-oriented systems are often characterized by following a well-defined problem structure, which is closely related to a specific domain. Task-oriented dialogue systems often need to query an external Knowledge Base (KB), which is used to complete complex tasks by reasoning through bodies of knowledge and fill the gap of background knowledge required for the conversation between humans and systems. Knowledge-based systems are designed to solve complex problems and even emulate the decision-making process of human experts by reasoning through bodies of knowledge. It gives an explanation capability in a similar way that human experts could explain the reasoning process. Another advantage is that the KBS can infer new facts from those known already, which reduces the number of turns necessary to meet user goals. The KBS sometimes will ask the user for further information that is used to guide the line of reasoning. The new facts inferred and the further information asked by the KBS may change the state of dialogue as well as the following system actions which adds complexity to the design of Expert System. A major advantage of KBS technology is that it can handle unexpected inputs deviating from a predefined pattern.

Deep neural networks have been demonstrated to be effective in capturing high-level complex features and patterns in many research fields including computer vision and natural language processing. Unlike the traditional pipeline solutions, recently proposed end-to-end (trainable) neural networks offer a promising solution to task-oriented dialogue systems. However, neural dialogue systems need to be trained on a large dialogue data annotated manually by humans. The amount of data for a specific domain is often insufficient because domain-specific data collection and annotation are expensive and time-consuming. Those systems are trained to speak by mimicking a response repeatedly many times for a given input, but the generated responses still lack naturalness and diversity and sometimes are not consistent and meaningful. Arguably, the biggest disadvantage of neural networks is their “black box” nature—it is hard to know how or why a neural network comes up with a certain output. The dialogue systems should be able to explain the reasoning behind their answers in the same way that a human expert can explain why a conclusion was reached, which makes them understandable and thus trustworthy to the users. It is very important for dialogue systems to have such features for several reasons. One reason is that the answers of a system may affect the health of human life or the safety of property. For example, in the medical domain, a user will not blindly trust a system that recommends an invasive surgery without giving its justification, because it may cause a serious negative impact. A system would be asked to justify its recommendations and explain the steps of reasoning. The second reason for possessing this explanation capability is to confirm that the knowledge has been accurately acquired and to provide the understandable check of reasoning about the acquired knowledge in the development stage of a task-oriented dialogue system. Rule-based systems, especially for those having their roots in a certain formal logic, are attractive for various tasks since they inherently can yield explainable and understandable decisions.

## 2 Literature survey

Humans are concerned with plant protection after the advent of agriculture. It all started when humans tried to comprehend agricultural diseases, which are today known as ‘abiotic’ and ‘biotic’ problems. Plants have been afflicted by bacteria, fungi, viruses, insects, and nematodes for millennia. When pests infested the first crop of cultivated plants, a critical step toward plant protection was taken. Time-tested traditional methods become more important when the effects of the so-called Green Revolution, which was primarily dependent on chemical inputs, begin to fade. Now we've moved on to genetically modified crops, with little regard for the long-term effects [5]. *Vrikshayurveda* text has twelve chapters viz. *Bhumi-nirupana* (Land Profile - soil classification), *Bijoptivithi* (process of seed germination), *Padapavivaksa*(Plant Profile - tree biology, plant have life and senses), *Ropana vidhana*(Plantation procedure-The procedure to facilitate wound healing for plants), *Nise canavidhi* (the life of plant), Posana vidhi (Nurture-Plant nourishment), *Drumaraksa* (Protection of plant), *Taru-Chikitsa* (Plant treatment), *Upavanakriya* (Gardening), *Nivasanna-taruropanam* (Plantation of tree near residential complex), *Subhasubha-Laksana* (auspicious and inauspicious symptoms), and *Taru Mahima-Citrikarana* (Glory of tree filming). *Vrikshayurveda* is also mentioned in the Brhatsamhita (authored by Varahamihira) from the sixth century [6,7]. It also includes chapters on related topics such as groundwater divination, land productivity and non-production as represented by natural vegetation, and so on. However, it cannot provide any solid hints to any full-fledged, independent literature on *Vrikshayurveda* beyond confirming the sastra's antiquity. Another ancient literature, Sarngadharapaddhati (authored by Sarngadhara), is an anthological compilation from the thirteenth century that deals with an analogous subject, “arbori-horticulture,” in its chapter “Upavanavinoda” [8]. Here arboriculture deals with the study of trees and horticulture deals with study of plants. The focus of *Vrikshayurveda* and *Krishishastra*, now that we have the original texts available with us, is on the field use of these prescriptions and practices to the extent possible. Agro traditional practices offer priceless strategies for preventing the development of plant pathogenic illnesses, maximizing the benefits, and improving soil fertility [9]. This would ensure reduction of use of chemicals and take our agriculture towards organic farming. This is to stimulate the young minds to undertake in-depth research in several topics in both *Vrikshayurveda* and *Krishishastra.* Automation to access the traditional Indian knowledge system with regard to flora and fauna is need of the hour.

## 3 Methodology

This work implements a diagnostic expert system in python to identify and categorize various disorders of trees based on their symptoms. It is implemented using decorators, and two library modules, that is, Experta and Flask. The expert system creates a hypothesis about the disorder in a hierarchical format. That is, the hypothesis categorizes different disorders under it. The hypothesis is further classified as internal and external, and each category has specific subcategories.

This code reflects an attempt to organize and classify disorders based on their symptoms. This approach can help in better understanding and categorization of disorders, which can be valuable in various fields such as healthcare, Ayurvedic medicine, and research.

By using the Experta module, one can utilize the logical programming paradigm to define relationships between different disorders and their classifications. Experta's ability to perform automated reasoning and logical inference can assist in querying and exploring the knowledge base to gain insights into various disorders and their categorization.

### 3.1 Problem statement

The problem addressed in this research is the diagnosis of disorders in plants using logic programming. The motivation behind this research stems from the effectiveness of Ayurvedic medicines and treatments in providing long-term health benefits to individuals.

The objective here is to create an expert system that can precisely identify disorders in plants based on observed symptoms and provide appropriate Ayurvedic treatments.

To provide the knowledge from *Vrikshayurveda* in an expert system form for easy accessibility and to enable reintroduction of superior organic farming techniques from Indian Knowledge System, specifically from *Vrikshayurveda*, for plant growth and conservation to the current generation.

To achieve this objective, Experta module is employed as the chosen methodology. Experta offers a declarative approach to problem-solving, where facts, rules, and queries are utilized to reason and make inferences. By leveraging the knowledge and expertise embedded in the rule-based system, the developed expert system can effectively identify disorders in plants and suggest corresponding Ayurvedic treatments.

### 3.2 Hypothesis formulation

The hypothesis Formulation involves identifying hypotheses about the presence of specific disorders in plants based on the observed symptoms. The disorders considered may include categories such as “*vata*”, “*pitta*”, “*kapha*” or other Ayurvedic classifications. Each disorder category is associated with a distinct set of symptoms. The hypothesis formulation process can be further elaborated as follows:

I. Identifying Hypotheses: The research identifies different disorder categories based on Ayurvedic principles and expert knowledge. For example, “*vata*” may be associated with symptoms such as dryness, roughness, and mobility issues, while “*pitta*” may manifest as symptoms like inflammation, heat, and irritability. Hypotheses are then identified for each disorder category.
II. Integration with Experta: The formulated hypotheses are integrated into the Experta program as predicates, allowing the system to reason about the presence of specific disorders based on observed symptoms.
III. Symptom Evaluation: The system prompts the user with a series of yes/no questions about the presence of symptoms in the plant. These questions correspond to the symptoms associated with each disorder category. The user's responses are recorded for further evaluation.
IV. Verification and Inference: The system evaluates the user's responses and checks whether the observed symptoms align with the symptoms associated with the hypothesized disorder category. It uses logical inference rules defined in Experta to reach conclusions about the presence or absence of the hypothesized disorder.
V. Updating Beliefs: Based on the evaluation and inference process, the system updates its belief statement regarding the presence of a specific disorder in the plant. If the observed symptoms match the symptoms associated with a particular disorder, the system concludes that the plant likely has that disorder.

### 3.3 Symptom collection and verification

Symptoms related to each disorder category are identified and collected. The user is prompted with yes/no questions regarding the presence of each symptom. The responses are used to verify the presence or absence of symptoms in the plant. The process of symptom collection and verification include the following steps as follows:

I. Identification of Symptoms: Based on Ayurvedic principles and expert knowledge, a list of symptoms is determined for each disorder category. These symptoms are indicative of specific disorders that can affect plants. For example, symptoms associated with “*vata*” disorder may include dryness, wilting, or stunted growth, while symptoms for “*pitta*” disorder may include yellowing leaves, burning sensation, or excessive wind.
II. Prompting User with Questions: The expert system prompts the user with a series of yes/no questions regarding the presence of each symptom. For example, the user may be asked, “Does the plant exhibit dryness in leaves?” or “Is there a burning sensation observed in the plant?” The questions are designed to gather information about the symptoms observed in the plant.
III. User Response and Verification: The user responds to each question with a “yes” or “no” answer, indicating the presence or absence of the symptom in the plant. The expert system records these responses for further evaluation.
IV. Matching Symptoms with Hypothesized Disorder: The recorded user responses are then matched with the symptoms associated with the hypothesized disorder. If the user confirms the presence of a symptom associated with the hypothesized disorder, it provides evidence in support of the hypothesis. Conversely, if the user denies the presence of a symptom associated with the hypothesized disorder, it suggests that the hypothesis is less likely.
V. Updating Beliefs: Based on the verification process, the expert system updates its belief statement regarding the presence or absence of each symptom in the plant. The belief statement is continuously revised as more symptoms are evaluated and verified.

### 3.4 Belief statement

The conclusion and belief statement phase are where the final determination regarding the disorder of the plant is made based on the verified symptoms. By analyzing the collected symptom data, a conclusion is reached regarding the specific disorder that best aligns with the observed symptoms. This conclusion serves as the basis for generating a belief statement, which clearly states the identified disorder of the plant. For example, if the symptoms align with the “vata” disorder, the belief statement would indicate that the plant has been classified as having “vata” disorder. The conclusion and belief statement provide a concise summary of the diagnostic outcome and contribute to the overall classification of the plant's disorder. Predicates are defined for hypothesis formulation, symptom verification, and belief generation. The code iteratively collects and verifies symptoms, updating the belief statement accordingly.

The work requires expertise in Sanskrit to interpret knowledge that is in a very concise form (Sutras) correctly with the help of experts in Agriculture; and convert that knowledge by a Computer Science expert to a form where computer program in form of an expert system can do inferences on user/stakeholder queries. We have used the translated version of Vrikshayurveda [1,2]. A web application is developed to key-in the user input. The output is displayed on the monitor.

**Fig. 2:**
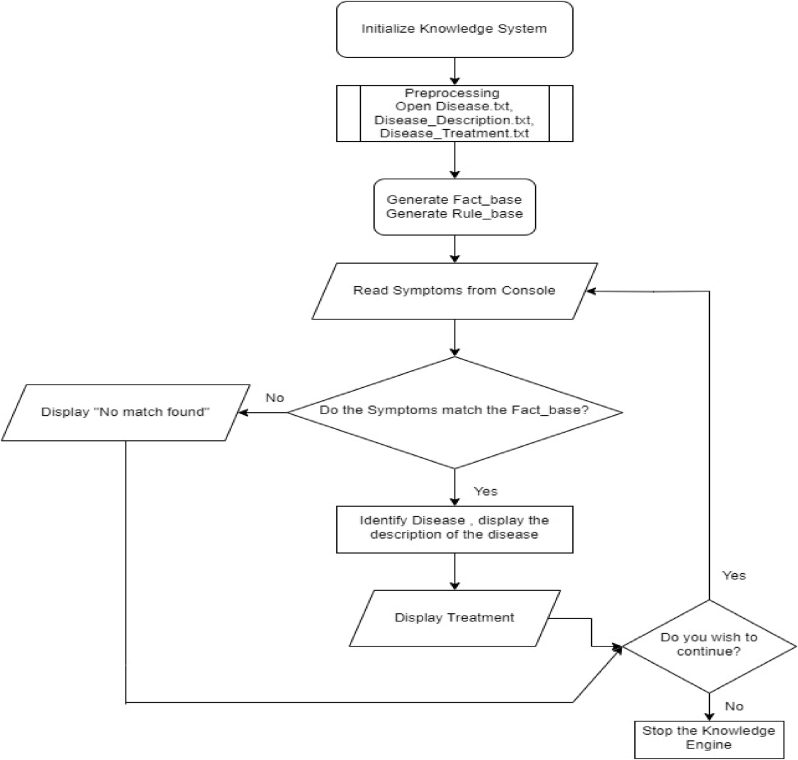
Flow diagram of Vrikshayurveda Expert System.

A rule based backward chaining expert system using Experta Python module is built [10,11]. The three databases “Disease Descriptions”, “Disease symptoms” and “Disease treatments” are created. The flow diagram is shown in Fig. 2.

## 4 Result

The developed system is evaluated using sample plant data and known disorders. The accuracy of the system in correctly classifying plant disorders is assessed. Validation techniques, such as cross-validation or expert evaluation, can be employed to ensure the system's reliability.

**Fig. 3:**
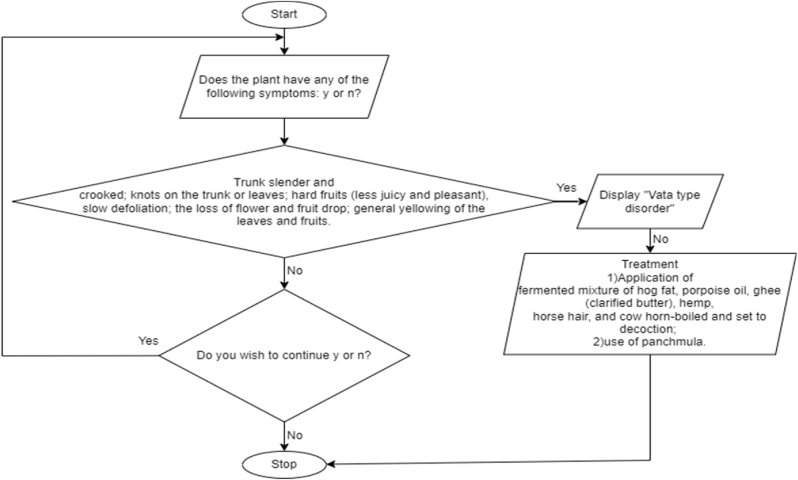
Flow diagram for Vata-type disorder.

The symptoms are not user-provided but are hardcoded within the program. The code follows a rule-based approach where it checks the presence of predefined symptoms for a particular disorder. If all the symptoms are answered as “yes” by the user, the code identifies the corresponding disorder. However, if any symptom is answered as “no,” the code does not accurately identify the disorder. The evaluation of this code would involve testing its performance on a set of predefined cases and comparing the results with the expected outcomes. The validation process would require expert evaluation to assess the accuracy of the code's classifications. It is important to acknowledge the limitations of the code, such as its reliance on hardcoded symptoms and the potential for inaccurate identification if a symptom is missed or answered incorrectly. Ten different types of diseases are tested using this application. Various literature were studied while developing this work as shown in appendix Table 1. The Flow diagram for Vata-type disorder is shown in Fig. 3.

## 5 Discussion

Preserving and getting benefited by ancient Indian knowledge with regard to Vrikshayurveda is the need of the hour. Western countries are preferring organic farming. Kunapajala, the fermented liquid manure used in ancient India, was a fantastic invention. Agronomists all over the world currently are inclined to the idea that the invention of fermented liquid manure was made by farmers in Japan, Korea, China, or even mediaeval Europe not by farmers of India because the fact that Kunapajala was Indian went unnoticed for centuries [12]. In this context government and agriculture sectors should promote Vrikshayurveda at bigger scale. Pantnagar University established by Dr. Y. L.Nene Pantnagar, Uttarakhand has included Vrikshayuveda syllabus in the curriculum.Project broader impacts are as follows:1) Sustainable vegetation techniques for the protection of Soil, plants and the whole ecosystem 2) Protection of native plant species rather than genetically modifies species 3) Healthy surroundings for farming 4) Fertilizers that are produced locally and naturally 5)Possibility of growing a range of crops.Therefore, farming based on Vrikshayurvedic theory holds the key to a happy, healthy, and tranquil life for people in the 21st century. Limitations: These ingredients - fermented mixture of hog fat, porpoise oil, ghee, hemp, horse hair, boiled cow horn, and punchmula - are challenging to source.

## 6 Conclusion

The world is turning its attention to traditional, conservative, and organic farming methods. The philosophy of Vrikshayurveda has traditionally emphasized prevention over cure, and it also makes several recommendations for maintaining biodiversity. The ancient Ayurvedic literature contains numerous recipes for organic pesticides and manures, and modern agricultural experts and research facilities are using these recipes with great success.

## Figures and Tables

**Table 1:** Important statistical data of the system

No. of sutras of Vrishayurveda studied	Foundation texts	No. of symptoms	No. of diseases	No. of treatment
Verse 101. The excreta, marrow of the bones, flesh, brain, and blood of a boar mixed with water and stored underground is called kunapa. Verse 102. As per availability, the fat, marrow, and the flesh of fish, the ram, the goat, and other horned animals should be collected and stored. Verse 103. These should be boiled after mixing with water, and the mixture should be stored in an oiled pot after adding sufficient quantity of husk. Verse 104. After roasting (cooking) it in an iron pot, sesame oil cake and honey should be added. Soaked black gram of good quality should also be added. A little ghee should then be poured. Verse 105. The items stated above should be taken at random as there is no measure for anything. One by one, items should be placed in the pot in a warm place by a competent person. Verse 106. This kunapa is highly nourishing for the trees. This is as stated by the ancient sages and Surapala Verse 185.Disease caused by imbalance of vata can be cured by flesh, marrow and ghee. Sprinkling of kunapa water also removes all the disorders caused by the vata element Verse 186. The diseases of vata type can be quickly warded off by the mixture of the fat of the hog, oil of the Gangetic porpoise, ghee, hemp, hair of the horses, and cow's horn-boiled and set to a decoction. Verse 187. The diseases of the kafa type can be overcome with bitter, strong, and astringent decoctions made out of panchamula (roots of five plant species – sriphala, sarvatobhadra, patala, ganikarika, and syonaka) with fragrant water. Verse 188. For warding off all kafa type of diseases, the paste of white mustard should be deposited at the root and the trees should be watered with a mixture of sesame and ashes. Verse 189. In case of trees affected by the kafa disease, earth around the roots of the trees should be removed and fresh, dry earth should be replaced for curing them. Verse 190. A wise person should treat all types of trees affected by the pitta type of diseases with cool and sweet substances. Verse 191. When watered by the decoction of milk, honey, yastimadhu, and madhuka, trees suffering from pitta type of diseases get cured. Verse 192. Watered with the decoctions of fruits, triphala, ghee, and honey the trees are freed of all diseases of the pitta type. Verse 193. To remove insects both from the roots and branches of the trees, wise men should water the trees with cold water for seven days. Verse 194. The worms can be overcome by the paste of milk, kunapa water, and cow dung mixed with water and also by smearing the roots with the mixture of white mustard, vaca, kusta, and ativisa. Verse 195. The worms accumulated on trees can be treated quickly by smoking the tree with the mixture of white mustard, ramatha, vidanga, vaca, usana, and water mixed with beef, horn of a buffalo, flesh of a pigeon, and the powder of bhillata (bhallataka ?). Verse 196. Anointing with vidanga mixed with ghee, watering for seven days with salt water, and (applying) ointment made out of beef, white mustard, and sesame destroy the worms, insects, etc. Verse 197. Creepers eaten away by insects should be sprinkled with water mixed with oil cake. The insects on the leaves can be destroyed by sprinkling the powder of ashes and brick-dust. Verse 198. A wound caused by insects heals if sprinkled with milk after being anointed with a mixture of vidanga, sesame, cow's urine, ghee, and mustard. Verse 199. Trees suffering from (damage due to) frost or scorching heat should be externally covered. Sprinkling with kunapa water and milk is also advisable. Verse 200–201. The broken trees should be smeared with the paste of the bark of plaksa and udumbara mixed with ghee, honey, wine, and milk and the broken parts should be firmly tied together with the rope of a rice stalk. Fresh soil should then be filled in the basin around the trees, sprinkled immediately with the milk of buffalo and flooded with water. Thus they recover. Verse 203. If the branches fall off, the particular spot should be anointed with the mixture of honey and ghee and sprinkled over by milk and water so that the tree will have its branches reaching the sky. Verse 204. If the branches are burnt they should be cut off and the particular spots should be sprinkled with water and milk and smoked with shells of crab, etc. Thus treated the trees will put forth sprouts. Verse 205. The trees are scorched with the fire the whole tree should be smeared with mud from the lotus creeper and then should be watered with kunapa mixture. Then its branches will grow up to sky Verse 206. When anointed with vidari, sugar, nagajivha (red arsenic), and sesame mixed together and when sprinkled with milk water, trees struck with lightning put forth healthy sprouts. Verse 207. Trees which are dried up due to heat caused by fire are cured when a mixture of sugar, sesame and milk is used for watering and anointing them and when they are smeared all around by mud from the bottom of a lotus plant Verse 208. If dried due to bad soil the original soil from the root should be removed and it should be replaced by healthy soil ad milk-water should be sprinkled over it. Verse 209. If the drying is due to the lack of water, the trees should be watered with milk-water and properly fomented by the smoke of crab shells. Verse 210. The wounds of trees are healed by the treatment of anointing with the paste of the bark of nyagrodha and udumbara, cow dung honey and ghee. Verse 211. The oozing can be cured by the (above stated) paste and by covering the pat with the barks of dhava, sripanika, syama and arjuna. Verse 212. Diseases caused by wrong treatment can be conquered by sprinkling the mixture of water and milk and also by applying a paste of vidanga mixed with thick mud. Verse 213. Jaundice can be brought under control only in weeks by sprinkling water mixed with the powder of barley and wheat added to honey and milk. Verse 214. Nonproductive trees bear fruits and flowers to one's complete satisfaction when they are fed with milk and kunapa water. Verse 215. Un productive trees fill the quarters with branches covered with flowers and fruits without fail if treated with coldmixture of sesame, barley, kulattha, green gram and black gram. Verse 216–217. Sesame and the dung of the goat and sheep each measuring one adhaka (256 handfuls), barley measuring one prastha 64 handfuls), water measuring one drona (1024 handfuls) and corresponding quantity of beef if allowed to set for seven nights and then used for watering, the trees put forth flowers and fruits. Verse 218. They produce fruits also if watered with the thick mixture of the flesh the tiger leopard and fox and with milk of elephant and buffalo Verse 219. Tender plants suffering from excess watering should be scratched with nails uprooted and every root should be smeared with the mixture of honey and vidanga and then should be watered. Verse 220. Vegetables of cucumber types get cured of diseases when smoked around by the bones of cow and dog mixed with excreta of cat. Verse 221. Very tender plants should not be expose to excessive smoking. Excessive smearing, although gentle also should be avoided by the wise. Verse 222. Plants which are not cured by any one of the various above-stated remedies should be transplanted at other special sites	Sadhale, Nalini. (Tr.) 1996. Surapala's Vrikshayurveda (The Science of Plant Life by Surapala). Agri-History Bulletin No. 1. Asian Agri-History Foundation, Secunderabad 500 009, India. 104 pp.	20	11	38
Verses 171–174. One should boil the flesh, fat, marrow of deer, pig, fish, sheep, goat, and rhinoceros in water, and when it is properly boiled one should put the mixture in an earthen pot and add into the compound milk, powders of sesame oil cake, black gram boiled in honey, the decoction of pulses, ghee, and hot water. There is no fixity as to the amount of these elements; when the said pot is put in a warm place for about a fortnight, the compound becomes what is called kunapa water (kunapajala), which is very nourishing for plants in general.	Majumdar, G.P. 1935. Upavana-Vinoda (A Sanskrit Treatise on Arbori-Horticulture). Indian Research Institute, Calcutta, India. 128 pp.			
Verse 2. Fat, marrow, skin, blood along with the marrow secretion of ram, sheep, deer, fish, and so on should be mixed with water and cooked on fire. When properly cooked, milk and cold water should be added. Verse 3. Oilcake of sesame, honey, and ghee should be added to the mixture and the pot should be removed from fire to be kept in a warm place for a fortnight. This liquid called “kunapa” is nourishing for trees.	Sadhale, Nalini. (Tr.) 2004. Vishvavallabha (Dear to the World: The Science of Plant Life). Agri-History Bulletin No. 5. Asian Agri-History Foundation, Secunderabad 500 009, India. 134 pp.	10	10	10
Valmiki reported excellent results when kunapajala was applied to mango and coconut. He further tried a “herbal kunapa” using naturally fallen sour mango fruits and soapnut (Sapindus emarginatus) and applied it on chili plants with excellent results. Valmiki also published a report (Ayangarya, 2004b) on “manujala” in which he used vegetable organic wastes and fermented those in human urine. He again observed excellent effects on the growth of several fruit and vegetable plants. Valmiki continued his experimentation with kunapajala in Arunachal Pradesh in Northeast India (Ayangarya, 2005). He developed “herbal kunapa” and called it Sasyagavya. With the help of staff at the Abali Tea Estate, Abali Village, Roing 792 110, Arunachal Pradesh, he used to produce 5000 to 10 000 L Sasyagavya everyday and apply it to the soil. Tea plants started looking healthy. He prepared kunapajala by fermenting aerobically safari fish (mentioned in Vrikshayurveda) in cow urine and sprayed tea bushes at 1 % concentration of the ferment, which he named Indsafari, to most effectively control the attack by tea mosquito, Helopeltis with 10-day interval schedule. Foliar sprays with Indsafari at 1 % concentration also controlled the loopers on shade trees commonly grown in tea gardens. Valmiki found Indsafari both insecticide and growth promoter. In addition, Valmiki prepared kunapajala from poultry (chicken) bird flesh and called it kukkutakunapa (kukkuta = chicken), and used it very effectively in increasing kiwifruit yield from 120 kg in November 2004 to 1700 kg in November 2005 (Ayangarya, 2006b). In 2006, Valmiki (Ayangarya, 2006a) reported formulation of “mushika kunapa” (mushika = rats), which was prepared by 2-week aerobic fermentation of cut pieces of captured rats in cow urine. After filtering the liquid, it was sprayed on tea bushes at 1 % concentration. Results were most promising. Around the same time Narayanan (2006), a social worker in Tamil Nadu (5/47B Soundaram Nagar, Ambathurai Village, Gandhigram PO, Dindigal District 624 302, Tamil Nadu) reported formulation of “rat gunapa” or “mushika kunapa”. The “rat gunapa” (Narayanan, 2006) was prepared by a 2- week fermentation of rat pieces, 5 kg cow dung, 3 L cow urine, 500 g sugar, and 250 g black gram and sesame. Later 1 L cow milk and 100 ml honey were added. Not only growth effects were reported but also the rats disappeared from sprayed fields.	Valmiki Sreenivasa Ayangarya (2004a), (Ayangarya, 2004b) on “manujala” Ayangarya, 2006a on “mushika kunapa” Sasyagavya (“herbal kunapa”) “Indsafari” “kukkutakunapa” “mushika kunapa”	6	6	6
Fertilizer cum natural pesticide made from fish and animal waste called KUNAPA JAL	This fertilizer was made and applied in the tea gardens of Assam, Darjeeling, dooars and the Nilgiris and in the coffee estates of Karnataka. Various pest such as red spider mite and helopeltis which the tea gardens were unable to eliminate using chemical methods, were eliminated effortlessly with kunapa jal within a few months. Moreover, the tea bushes produced more green leaf and the fertility of the soil also increased as indicated by the return of earthworms in the soil. Laboratory soil tests showed that pesticide residues had been eliminated from the soil of these gardens within 4–6 months of steady and regular application of kunapa jal and other liquid manures.	2	2	2
Kunapa jal uses fish and animal waste and mustard oilcake; sasyagavya uses green weeds and cowdung; amritapani uses cowdung and jaggery; bhasmapani uses wood ash and cow urine; jaivik tika uses cowdung and cow urine; agnihotra bhasma is the ash obtained after performing agnihotra havan. All these manures can be made in simple plastic buckets or plastic drums or in cement tanks. The ingredients used in these manures are available locally and cheaply in the northeast. Most of these manures take from three to twenty days to be ready for field application only kunapa jal takes between 45 and 60 days to be ready in the plains.	Paddy farmers and small tea growers in the Golaghat area were also encouraged to prepare and use kunapa jal and other liquid manures in their gardens and fields. Dr. Padmeswar Gogoi a retired botanist has also praised these vrikshayurveda manures when he saw their wonderful field results. He is totally convinced about the efficacy of sasyagavya which uses green weeds and cowdung as raw materials. He has now become a champion of vrikshayurveda in Assam.			
Dr. Anjali Pathak is a naturopath, writer and organic farming consultant who has worked with the growers and the planters of the northeast, the dooars and the Nilgiris. She uses indigenous methods including those of vrikshayurveda in her work. She advises growers and conducts practical workshops on vrikshayurveda methods all over India www.naturalorganicfarming.com.	“Annam Brahma, Organic Food in India: Growing, Selling and Eating” Dr. Anjali Pathak			
kunapa jal and sasyagavya	The small tea farmers of Golaghat have demonstrated that the methods of vrikshayurveda are suited to tea, paddy and vegetable farming. They have achieved excellent results within a short period of time with only a modest financial investment. Their gardens and farms are now totally organic and some have been certified as organic. Apart from tea and paddy, the northeast is suited for the cultivation of various fruits and vegetables. Bhut jolokia has been in the news as the hottest chili in the world. Small growers are trying their best to grow bhut jolokia in their backyards and sell it at a handsome price. Similarly banana growers are making efforts to step up banana production in Assam. Keeping in mind the requirements of these small requirements, one big models have been developed for a few selected horticultural crops using the methods of vrikshayurveda for a bountiful harvest.			
A field trial on effect of herbal Kunapjala in Jasmine was conducted During 2017. A basal Application of 2L per plant, followed by Foliar application @ 200 ml/L of water at fortnightly intervals were applied. Soil acidity was corrected with lime Application @ 2kg/cent (40 square meter area or 500 kg/ha), before the start of the study. After three months of application, the farmer indicated that the floral buds were formed profusely, and aroma, appearance and shelf life of buds increased. Herbal Kunapjala was applied through fertigation and Cow urine based bio-pesticide as foliar application on Cowpea, Bhindi, Chilli, and Tomato. Farmers Were convinced about The effect of Herbal Kunapjala in Improving crop health, Higher yield and betterQuality of the produce. They were also convinced about the effect of Cow urine based bio-pesticide in managing caterpillars as well as sucking pests of these crops. After finishing the first barrel they prepared second and third barrel of 100 L each of herbal Kunapjala for application and they also supplied to farmers of other areas.	The popularization of Vrikshayurveda in Kannur District was done by organizing trainings at the Regional Agricultural Technology Training Centre (RATTC).			
Kunapa Jalam is recommended for stimulating growth and development of plants. Various parameters like plant height, leaf length, leaf number and inflorescence length were evaluated in test culture receiving Kunapa Jalam at different time intervals. Administration of Kunapa Jalam every tenth and fifteenth day exhibited remarkable enhancement in paddy growth. Further investigation in this direction will be important in the field of agriculture and can be a good substitute of synthetic fertilizer.	P K Mishra (May 2006) “Effect of Kunapa Jalam Vrikshyurveda on growth of paddy”			
Combination of both panchagavya and Kunapajala showed best results in influencing all the growth parameters as well as yield of the vegetables. Leaf chlorophyll content was also consistently higher by the application of two organics and that was ultimately reflected on enhanced fruit yield of the crops.	S Sarkar, SS Kundu & D Ghorai “Validation of ancient liquid organics - Panchagavya and Kunapajala as plant growth promoters “			
He applied Kunapajala regularly at 10–15 days interval in every crop and got amazingly good performance in every crop. He has harvested good quality bumper yields from pea, onion, marigold, tomato.	Mr Ranjeet Singh Bisht “Use of Herbal Kunapjala: A key for success of an innovative farmer of district Almora”			
The group tried Kunapajala on their onion and garlic crops. Prior to transplanting their onion seedlings they dipped their roots in Kunapajala and then applied Kunapajala at 15 days interval. Good onion yield (1.5 quintal from 1/2 nali, i.e., 100m^2^ area) and garlic yield (30 kg out of 4x4 sq meter area plot) were obtained.	Hansi devi Negi, Adhyaksha (Chairperson) of Mahila Mangal Dal at village Lodh, block Takula, district Almora, “Exploring Livelihood Potential of wild stinging nettle (Uritica dioica)” in Uttarakhand funded by Ministry of Environment, Forest and climate change Government of India under National Mission of Himalayan Studies (NMHS).			
In the 2020 crop season (March–October), a field experiment was conducted in a randomized block design with three replications to evaluate the efficacy of chemical fungicides, bio-agents and herbal Kunapajal. The experiment comprised of 13 treatments and an untreated control with 5 × 5 m plot size with nine fungicides. Application of Kunapajal found most effective in promoting plant growth parameters (e.g., plant height, number of tillers and number leaves).Herbal Kunapajala was also found effective in reducing disease severity to 66.21 % over the control plots.	Dr Laxmi Rawat, Asst Professor et. Uttarakhand University of Horticulture and Forestry, Bharsar “Usefulness of Herbal Kunapajala in managing stem (rhizome) rot of ginger”			
