# Supplementary material for: Technology from traditional knowledge - Vrikshayurveda-based expert system for diagnosis and management of plant diseases
Source: J Ayurveda Integr Med. 2024 Jan 13;15(1):100853. doi: 10.1016/j.jaim.2023.100853 (PMC10825595; doi:10.1016/j.jaim.2023.100853)
Supplement: Multimedia component 1 [file mmc1.docx]

. **Algorithm:** The Inference Algorithm

------------------------------------------------------------------------------------------------------------------------

**Require:**

A goal Q to be proved.

A knowledge base K which tells about the facts.

1. If (internal vata)

If ((trunk slender and crooked) and(knots on trunk or leaves) and(hard fruits less juicy and sweet) and(gradual defoliation) and(flower and fruit drop) and(general yellowing of leaves and fruits) and(arid land on account of excessive supply of dry and pungent matters) and(underground mechanical barrier) and(root infecting fungi or nematodes) and(viruses) and(saline or alkaline soils))

Print (1: Application of fermented mixture of hog fat, 2: porpoise oil, ghee (clarified butter), hemp, horsehair, and cow horn-boiled and set to decoction, 3: use of panchmula)

1. Else If (internal kapha)

If ((fruit bearing delayed and fruits are tasteless and ripened prematurely) and (oozing without wounds) and (appear in winter and spring if trees are excessively watered with sweet) and (oily sour or cold materials) and (fungal gummosis or rot) and (nutrient deficiencies or toxicities) and (excessive watering))

Print (1: Deposition of white mustard paste at the roots followed by watering trees with mixture of sesame and ash, 2: earth at the roots of trees should be removed and fresh dry earth should be placed)

1. Else if (internal pitta)

If ((leaf yellowing) and (premature drop) and (decay or flowers and fruits) and (occur at the end of the summer if trees are excessively watered with bitter sour salty and strong materials) and (viral disease) and (salinity in irrigation water) and (predisposal to blossom blight and fruit decays due to fungal bacterial infections))

Print (1: Watering trees with the decoction of milk, honey, yastamdhu and madhuka, 2: watering with decoction of fruits (triphala), ghee and honey)

1. Else if (external scorching heat)

If ((leaf yellowing) and (symptoms can be similar to vata type) and (roots eaten by insects) and (water stress pre-disposing trees to attack by pathogens or insects) and (frost damage))

Print(1:Insects on tree roots and branches can be removed by watering trees with cold water for seven days, 2:rubbing the roots with a mixture of white mustard, vasa, kusta, and ativisa, or with a paste made of milk, kunapa water, and cow dung with water, 3:milk is sprinkled over an insect-related wound before an application paste made of vidanga, 4:sesame, cow urine, ghee, and mustard is applied, 5:Covering trees and showering them with milk and kunapa water can help lessen the effects of freezing temperatures or intense heat)

1. Else if (external frost)

If ((leaf yellowing) and (symptoms can be similar to vata type) and (roots eaten by insects) and (water stress pre-disposing trees to attack by pathogens or insects) and (frost damage))

Print(1:Insects on tree roots and branches can be removed by watering trees with cold water for seven days, 2:rubbing the roots with a mixture of white mustard, vasa, kusta, and ativisa, or with a paste made of milk, kunapa water, and cow dung with water, 3:milk is sprinkled over an insect-related wound before an application paste made of vidanga, 4:sesame, cow urine, ghee, and mustard is applied, 5:Covering trees and showering them with milk and kunapa water can help lessen the effects of freezing temperatures or intense heat)

1. Else if (external excessive stormy winds)

If ((uprooting breaking of branches or twisting of trees) and (stormy winds) and (storm leading to mechanical damage))

Print(1:Insects on tree roots and branches can be removed by watering trees with cold water for seven days, 2:rubbing the roots with a mixture of white mustard, vasa, kusta, and ativisa, or with a paste made of milk, kunapa water, and cow dung with water, 3:milk is sprinkled over an insect-related wound before an application paste made of vidanga, 4:sesame, cow urine, ghee, and mustard is applied, 5:Covering trees and showering them with milk and kunapa water can help lessen the effects of freezing temperatures or intense heat)

1. Else

Print(Symptoms are not matching)

-----------------------------------------------------------------------------------------------------------------------------------

Sample Knowledge Base ( fact base + rule base)

/*Hypothesis that should be tested*/

hypothesis(internal_vata) :- internal_vata, !.

hypothesis(internal_kafa) :- internal_kafa, !.

hypothesis(internal_pitta) :- internal_pitta, !.

hypothesis(external_scorching_heat) :- external_scorching_heat, !.

hypothesis(external_frost) :- external_frost, !.

hypothesis(external_excessive_stormy_winds) :- external_excessive_stormy_winds, !.

hypothesis(unknown). /*no diagnosis */

/*Hypothesis Identification Rules*/

internal_vata:-

verify(trunk_slender_and_crooked),

verify(knots_on_trunk_or_leaves),

verify(hard_fruits_less_juicy_and_sweet),

verify(gradual_defoliation),

verify(flower_and_fruit_drop),

verify(general_yellowing_of_leaves_and_fruits),

verify(arid_land_on_account_of_excessive_supply_of_dry_and_pungent_matters),

verify(underground_mechanical_barrier),

verify(root_infecting_fungi_or_nematodes),

verify(viruses),

verify(saline/alkaline_soils),

write('Treatment'),

write('1:Application of fermented mixture of hog fat'),

write('2:porpoise oil, ghee (clarified butter), hemp, horsehair, and cow horn-boiled and set to decoction'),

write('3:use of panchmula')

-----------------------------------------------------------------------------------------------------------------------------------
