# Supplementary material for: Technology from traditional knowledge - Vrikshayurveda-based expert system for diagnosis and management of plant diseases
Source: J Ayurveda Integr Med. 2024 Jan 13;15(1):100853. doi: 10.1016/j.jaim.2023.100853 (PMC10825595; doi:10.1016/j.jaim.2023.100853)
Supplement: Multimedia component 2 [file mmc2.docx]

**Table 2. Information contained in the Vrikshyayurveda related to kinds of disorders in trees** [1, 2].

| **Disorder** | **Cause given** | **Symptoms** | **Cause elaborated** | **Possible causes** | **Treatment material** |
| --- | --- | --- | --- | --- | --- |
| Internal | Vata | Trunk slender and crooked; knots on the trunk or leaves; hard fruits (less juicy and pleasant), slow defoliation; the loss of flower and fruit drop; general yellowing of the leaves and fruits. | Arid land | Root infecting fungi or nematodes; viruses; saline/ alkaline soils. | Application of fermented mixture of hog fat, porpoise oil, ghee (clarified butter), hemp, horse hair, and cow horn-boiled and set to decoction; also use of panchmula. |
|  | Kapha | Fruit bearing is delayed, and the fruits are bland and overripe; oozing without injuries. | Appear in winter and spring | Fungal gummosis/rot; nutrient deficiencies or toxicities; excessive watering. | Application of white mustard paste at the roots, followed by the watering of the trees with a sesame-and-ash mixture; the earth at the roots of the trees should then be removed and replaced with fresh, dry earth. |
|  | Pitta | Early leaf withering; Premature drop; fruit or flower decay. | Occur at the end of the summer. | Viral disease; salinity in irrigation water; Predisposal to blossom blight and fruit decays due to fungal/ bacterial infections. | Watering trees with a decoction of triphala, ghee, and honey; and watering with a decoction of milk, honey, yastamdhu, and madhuka. |
| External | Scorching heat/Frost | Leaf yellowing; vata-like symptoms may be present. | Roots eaten by insects. | Water stress pre-disposing trees to attack by pathogens/insects, frost damage | Insects can be removed by cold water for seven days; rubbing the roots with a mixture of white mustard, vasa, kusta, and ativisa, or with a paste made of milk, kunapa water, and cow dung with water; milk is sprinkled over an insect-related wound before an application paste made of vidanga, |
|  | Excessive stormy winds | Tree uprooting, branch breaking, or tree twisting. | stormy winds | Strom leading to mechanical damage | Broken trees: Prepare a mixture of the bark of Plaksa (Ficus lacor), Udumbara (Ficus glomerata) ghee, honey, wine and milk and apply to the broken parts of the tree. |
|  | Fire / lightning, Soil aridity, Water Stress | Drying of trees and vata-like symptoms if exposed to heat on arid soils. | NA | Same as causes given. | Burned branches should be removed, and the cuts should be moistened with milk and water before being smoked with crab shells. If burned, the entire tree should be covered in lotus mud before being watered with a kunapa mixture. Trees struck by lightning need to be anointed with a mixture of vidari, sugar, red arsenic, and sesame, then sprinkled with milk and water. Water-stress is caused by the combination of milk water and crab shell smoke. |
|  | Struck by axe etc | Trees wounded resulting in drying up | NA | Same as causes given. | Tree wounds are healed if paste of the bark of nyagrodha (Banyan tree) and udumbara (fig tree), cow dung, honey and ghee is applied. |
|  | Faulty Seed | Trees become unproductive | Lack of appropriate seed treatment; wrong remedies used | Seed infected with pathogens or infected by insects. | Seed should be treated with milk, mustard, ash of sesame and brhati, rubbing with cow dung, honey and / or bidanga. |
|  | Ants | Foul smell, original fragrance missing; reduction of leaf size, stunted seedlings | NA | Ants could mean a wide range of insects. | Worms (caterpillers on trees can be removed by smoking with a mixture of white mustard, ramatha, vidanga, vaca, usana and water mixed with beef, pigeon flesh, billatta powder, and horn of a buffalo. Trees can also be anointed with vidanaga mixed with ghee, watered for seven days with soft water, and treated with an ointment made of beef. |
|  | Excessive watering | Symptoms similar to those produced in case of damage by ants.  Destruction of trees; | Trees suffer from indigestion. | Trees suffer from indigestion. | A mixture of honey and vidanga should be applied to every root of tender plants after being uprooted and scratched with nails. |
|  | Friction with other trees, continuous shade, inhabitation by too many birds, excessive growth of creepers, growth of weeds nearby. | Destuction of trees | NA | NA | Manuscript is silent; perhaps it is assumed that causes will be eliminated by farmers. |
