# Supplementary material for: Technology from traditional knowledge - Vrikshayurveda-based expert system for diagnosis and management of plant diseases
Source: J Ayurveda Integr Med. 2024 Jan 13;15(1):100853. doi: 10.1016/j.jaim.2023.100853 (PMC10825595; doi:10.1016/j.jaim.2023.100853)
Supplement: Multimedia component 3 [file mmc3.docx]

1. **Appendix**

Table 1. Important statistical data of the system

| No. of sutras of Vrishayurveda studied | Foundation texts | No. of symptoms | No. of diseases | No. of treatment |
| --- | --- | --- | --- | --- |
| Verse 101. The excreta, marrow of the bones, flesh, brain, and blood of a boar mixed with water and stored underground is called kunapa.  Verse 102. As per availability, the fat, marrow, and the flesh of fish, the ram, the goat, and other horned animals should be collected and stored.  Verse 103. These should be boiled after mixing with water, and the mixture should be stored in an oiled pot after adding sufficient quantity of husk.  Verse 104. After roasting (cooking) it in an iron pot, sesame oil cake and honey should be added. Soaked black gram of good quality should also be added. A little ghee should then be poured.  Verse 105. The items stated above should be taken at random as there is no measure for anything. One by one, items should be placed in the pot in a warm place by a competent person.  Verse 106. This kunapa is highly nourishing for the trees. This is as stated by the ancient sages and Surapala  Verse 185.Disease caused by imbalance of vata can be cured by flesh, marrow and ghee. Sprinkling of kunapa water also removes all the disorders caused by the vata element  Verse 186. The diseases of vata type can be quickly warded off by the mixture of the fat of the hog, oil of the Gangetic porpoise, ghee, hemp, hair of the horses, and cow’s horn-boiled and set to a decoction.  Verse 187. The diseases of the kafa type can be overcome with bitter, strong, and astringent decoctions made out of panchamula (roots of five plant species – sriphala, sarvatobhadra, patala, ganikarika, and syonaka) with fragrant water.  Verse 188. For warding off all kafa type of diseases, the paste of white mustard should be deposited at the root and the trees should be watered with a mixture of sesame and ashes.  Verse 189. In case of trees affected by the kafa disease, earth around the roots of the trees should be removed and fresh, dry earth should be replaced for curing them.  Verse 190. A wise person should treat all types of trees affected by the pitta type of diseases with cool and sweet substances.  Verse 191. When watered by the decoction of milk, honey, yastimadhu, and madhuka, trees suffering from pitta type of diseases get cured.  Verse 192. Watered with the decoctions of fruits, triphala, ghee, and honey the trees are freed of all diseases of the pitta type.  Verse 193. To remove insects both from the roots and branches of the trees, wise men should water the trees with cold water for seven days.  Verse 194. The worms can be overcome by the paste of milk, kunapa water, and cow dung mixed with water and also by smearing the roots with the mixture of white mustard, vaca, kusta, and ativisa.  Verse 195. The worms accumulated on trees can be treated quickly by smoking the tree with the mixture of white mustard, ramatha, vidanga, vaca, usana, and water mixed with beef, horn of a buffalo, flesh of a pigeon, and the powder of bhillata (bhallataka ?).  Verse 196. Anointing with vidanga mixed with ghee, watering for seven days with salt water, and (applying) ointment made out of beef, white mustard, and sesame destroy the worms, insects, etc.  Verse 197. Creepers eaten away by insects should be sprinkled with water mixed with oil cake. The insects on the leaves can be destroyed by sprinkling the powder of ashes and brick-dust.  Verse 198. A wound caused by insects heals if sprinkled with milk after being anointed with a mixture of vidanga, sesame, cow's urine, ghee, and mustard.  Verse 199. Trees suffering from (damage due to) frost or scorching heat should be externally covered. Sprinkling with kunapa water and milk is also advisable.  Verse 200-201. The broken trees should be smeared with the paste of the bark of plaksa and udumbara mixed with ghee, honey, wine, and milk and the broken parts should be firmly tied together with the rope of a rice stalk. Fresh soil should then be filled in the basin around the trees, sprinkled immediately with the milk of buffalo and flooded with water. Thus they recover.  Verse 203. If the branches fall off, the particular spot should be anointed with the mixture of honey and ghee and sprinkled over by milk and water so that the tree will have its branches reaching the sky.  Verse 204. If the branches are burnt they should be cut off and the particular spots should be sprinkled with water and milk and smoked with shells of crab, etc. Thus treated the trees will put forth sprouts.  Verse 205. The trees are scorched with the fire the whole tree should be smeared with mud from the lotus creeper and then should be watered with kunapa mixture. Then its branches will grow up to sky  Verse 206. When anointed with vidari, sugar, nagajivha(red arsenic), and sesame mixed together and when sprinkled with milk water, trees struck with lightning put forth healthy sprouts.  Verse 207. Trees which are dried up due to heat caused by fire are cured when a mixture of sugar, sesame and milk is used for watering and anointing them and when they are smeared all around by mud from the bottom of a lotus plant  Verse 208. If dried due to bad soil the original soil from the root should be removed and it should be replaced by healthy soil ad milk-water should be sprinkled over it.  Verse 209. If the drying is due to the lack of water, the trees should be watered with milk-water and properly fomented by the smoke of crab shells.  Verse 210. The wounds of trees are healed by the treatment of anointing with the paste of the bark of nyagrodha and udumbara, cow dung honey and ghee.  Verse 211. The oozing can be cured by the (above stated) paste andby covering the pat with the barks of dhava, sripanika, syama and arjuna.  Verse 212. Diseases caused by wrong treatment can be conquered by sprinkling the mixture of water and milk and also by applying a paste of vidanga mixed with thick mud.  Verse 213. Jaundice can be brought under control only in weeks by sprinkling water mixed with the powder of barley and wheat added to honey and milk.  Verse 214. Nonproductive trees bear fruits and flowers to one’s complete satisfaction when they are fed with milk and kunapa water.  Verse 215. Un productive trees fill the quarters with branches covered with flowers and fruits without fail if treated with coldmixture of sesame, barley , kulattha, green gram and black gram.  Verse 216-217. Sesame and the dung of the goat and sheep each measuring one adhaka(256 handfuls), barley measuring one prastha 64 handfuls), water measuring one drona( 1024 handfuls) and corresponding quantity of beef if allowed to set for seven nights and then used for watering, the trees put forth flowers and fruits.  Verse 218. They produce fruits also if watered with the thick mixture of the flesh the tiger leopard and fox and with milk of elephant and buffalo  Verse 219. Tender plants suffering from excess watering should be scratched with nails uprooted and every root should be smeared with the mixture of honey and vidanga and then should be watered.  Verse 220. Vegetables of cucumber types get cured of diseases when smoked around by the bones of cow and dog mixed with excreta of cat.  Verse 221. Very tender plants should not be expose to excessive smoking. Excessive smearing, although gentle also should be avoided by the wise.  Verse 222. Plants which are not cured by any one of the various above-stated remedies should be transplanted at other special sites | Sadhale, Nalini. (Tr.) 1996. Surapala’s Vrikshayurveda (The Science of Plant Life by Surapala). Agri-History Bulletin  No. 1. Asian Agri-History Foundation, Secunderabad 500 009, India. 104 pp. | 20 | 11 | 38 |
| Verses 171–174. One should boil the flesh, fat, marrow of deer, pig, fish, sheep, goat, and rhinoceros in water, and when it is properly boiled one should put the mixture in an earthen pot and add into the compound milk, powders of sesame oil cake, black gram boiled in honey, the decoction of pulses, ghee, and hot water. There is no fixity as to the amount of these elements; when the said pot is put in a warm place for about a fortnight, the compound becomes what is called kunapa water (kunapajala), which is very nourishing for plants in general. | Majumdar, G.P. 1935. Upavana-Vinoda (A Sanskrit Treatise on Arbori-Horticulture). Indian Research Institute, Calcutta,  India. 128 pp. |  |  |  |
| Verse 2. Fat, marrow, skin, blood along with the marrow secretion of ram, sheep, deer, fish, and so on should be mixed with water and cooked on fire. When properly cooked, milk and cold water should be added.  Verse 3. Oilcake of sesame, honey, and ghee should be added to the mixture and the pot should be removed from fire to be kept in a warm place for a fortnight. This liquid called “kunapa” is nourishing for trees. | Sadhale, Nalini. (Tr.) 2004. Vishvavallabha (Dear to the World: The Science of Plant Life). Agri-History Bulletin No. 5.  Asian Agri-History Foundation, Secunderabad 500 009, India. 134 pp. | 10 | 10 | 10 |
| Valmiki reported excellent results when kunapajala was applied to mango and coconut. He further tried a “herbal kunapa” using naturally fallen sour mango fruits and soapnut (Sapindus emarginatus) and applied it on chili plants with excellent results. Valmiki also published a report (Ayangarya, 2004b) on “manujala” in which he used vegetable organic wastes and fermented those in human urine. He again observed excellent effects on the growth of several fruit and vegetable plants. Valmiki continued his experimentation with kunapajala in Arunachal Pradesh in Northeast India (Ayangarya, 2005). He developed “herbal kunapa” and called it Sasyagavya. With the help of staff at the Abali Tea Estate, Abali Village, Roing 792 110, Arunachal Pradesh, he used to produce 5,000 to 10,000 liters Sasyagavya everyday and apply it to the soil. Tea plants started looking healthy. He prepared kunapajala by fermenting aerobically safari fish (mentioned in Vrikshayurveda) in cow urine and sprayed tea bushes at 1% concentration of the ferment, which he named Indsafari, to most effectively control the attack by tea mosquito, Helopeltis with 10-day interval schedule. Foliar sprays with Indsafari at 1% concentration also controlled the loopers on shade trees commonly grown in tea gardens. Valmiki found Indsafari both insecticide and growth promoter. In addition, Valmiki prepared kunapajala from poultry (chicken) bird flesh and called it kukkutakunapa (kukkuta=chicken), and used it very effectively in increasing kiwifruit yield from 120 kg in November 2004 to 1700 kg in November 2005 (Ayangarya, 2006b). In 2006, Valmiki (Ayangarya, 2006a) reported formulation of “mushika kunapa” (mushika=rats), which was prepared by 2-week aerobic fermentation of cut pieces of captured rats in cow urine. After filtering the liquid, it was sprayed on tea bushes at 1% concentration. Results were most promising. Around the same time Narayanan (2006), a social worker in Tamil Nadu (5/47B Soundaram Nagar, Ambathurai Village, Gandhigram PO, Dindigal District 624 302, Tamil Nadu) reported formulation of “rat gunapa” or “mushika kunapa”. The “rat gunapa” (Narayanan, 2006) was prepared by a 2- week fermentation of rat pieces, 5 kg cow dung, 3 L cow urine, 500 g sugar, and 250 g black gram and sesame. Later 1 L cow milk and 100 ml honey were added. Not only growth effects were reported but also the rats disappeared from sprayed fields. | Valmiki Sreenivasa Ayangarya Ayangarya(2004a), (Ayangarya, 2004b) on “manujala”  Ayangarya, 2006a on “mushika kunapa”  Sasyagavya(“herbal kunapa”)  “Indsafari”  “kukkutakunapa”  “mushika kunapa” | 6 | 6 | 6 |
| Fertilizer cum natural pesticide made from fish and animal waste called KUNAPA JAL | This fertilizer was made and applied in the tea gardens of Assam, Darjeeling, dooars and the Nilgiris and in the coffee estates of Karnataka. Various pest such as red spider mite and helopeltis which the tea gardens were unable to eliminate using chemical methods, were eliminated effortlessly with kunapa jal within a few months. Moreover, the tea bushes produced more green leaf and the fertility of the soil also increased as indicated by the return of earthworms in the soil.  Laboratory soil tests showed that pesticide residues had been eliminated from the soil of these gardens within 4-6 months of steady and regular application of kunapa jal and other liquid manures. | 2 | 2 | 2 |
| Kunapa jal uses fish and animal waste and mustard oilcake; sasyagavya uses green weeds and cowdung; amritapani uses cowdung and jaggery; bhasmapani uses wood ash and cow urine; jaivik tika uses cowdung and cow urine; agnihotra bhasma is the ash obtained after performing agnihotra havan.  All these manures can be made in simple plastic buckets or plastic drums or in cement tanks. The ingredients used in these manures are available locally and cheaply in the northeast.  Most of these manures take from three to twenty days to be ready for field application only kunapa jal takes between 45-60 days to be ready in the plains. | Paddy farmers and small tea growers in the Golaghat area were also encouraged to prepare and use kunapa jal and other liquid manures in their gardens and fields. Dr. Padmeswar Gogoi a retired botanist has also praised these vrikshayurveda manures when he saw their wonderful field results. He is totally convinced about the efficacy of sasyagavya which uses green weeds and cowdung as raw materials. He has now become a champion of vrikshayurveda in Assam. |  |  |  |
| Dr. Anjali Pathak is a naturopath, writer and organic farming consultant who has worked with the growers and the planters of the northeast, the dooars and the Nilgiris.  She uses indigenous methods including those of vrikshayurveda in her work. She advises growers and conducts practical workshops on vrikshayurveda methods all over India www.naturalorganicfarming.com. | “Annam Brahma, Organic Food in India: Growing, Selling and Eating” Dr. Anjali Pathak |  |  |  |
| kunapa jal and sasyagavya | The [small tea farmers](https://www.assamicaagro.in/pages/small-tea-growers-assam) of Golaghat have demonstrated that the methods of vrikshayurveda are suited to tea, paddy and vegetable farming. They have achieved excellent results within a short period of time with only a modest financial investment. Their gardens and farms are now totally organic and some have been certified as organic.  Apart from tea and paddy, the northeast is suited for the cultivation of various fruits and vegetables.  Bhut  jolokia has been in the news as the hottest chili in the world.  Small growers are trying their best to grow bhut jolokia in their backyards and sell it at a handsome price.  Similarly banana growers are making efforts to step up banana production in Assam.  Keeping in mind the requirements of these small requirements, one big models have been developed for a few selected horticultural crops using the methods of vrikshayurveda for a bountiful harvest. |  |  |  |
| A field trial on effect of herbal Kunapjala in Jasmine was conducted During 2017. A basal Application of 2L per plant, followed by Foliar application @ 200ml/L of water at fortnightly intervals were applied. Soil acidity was corrected with lime Application @ 2kg/cent (40 square meter area or 500 kg/ ha), before the start of the study. After three months of application, the farmer indicated that the floral buds were formed profusely, and aroma, appearance and shelf life of buds increased. Herbal Kunapjala was applied through fertigation and Cow urine based bio-pesticide as foliar application on Cowpea, Bhindi, Chilli, and Tomato. Farmers Were convinced about The effect of Herbal Kunapjala in Improving crop health, Higher yield and betterQuality of the produce. They were also convinced about the effect of Cow urine based bio-pesticide in managing caterpillars as well as sucking pests of these crops. After finishing the first barrel they prepared second and third barrel of 100 L each of herbal Kunapjala for application and they also supplied to farmers of other areas. | The popularization of Vrikshayurveda in Kannur District was done by organizing trainings at the Regional Agricultural Technology Training Centre (RATTC). |  |  |  |
| Kunapa Jalam is recommended for stimulating growth and development of plants. Various parameters like plant height, leaf length, leaf number and inflorescence length were evaluated in test culture receiving Kunapa Jalam at different time intervals. Administration of Kunapa Jalam every tenth and fifteenth day exhibited remarkable enhancement in paddy growth. Further investigation in this direction will be important in the field of agriculture and can be a good substitute of synthetic fertilizer. | P K Mishra (May 2006) “Effect of Kunapa Jalam Vrikshyurveda on growth of paddy” |  |  |  |
| Combination of both panchagavya and Kunapajala showed best results in influencing all the growth parameters as well as yield of the vegetables. Leaf chlorophyll content was also consistently higher by the application of two organics and that was ultimately reflected on enhanced fruit yield of the crops. | S Sarkar, SS Kundu & D Ghorai “Validation of ancient liquid organics - Panchagavya and Kunapajala as plant growth promoters “ |  |  |  |
| He applied Kunapajala regularly at 10-15 days interval in every crop and got amazingly good performance in every crop. He has harvested good quality bumper yields from pea, onion, marigold, tomato. | Mr Ranjeet Singh Bisht “Use of Herbal Kunapjala: A key for success of an innovative farmer of district Almora” |  |  |  |
| The group tried Kunapajala on their onion and garlic crops. Prior to transplanting their onion seedlings they dipped their roots in Kunapajala and then applied Kunapajala at 15 days interval. Good onion yield (1.5 quintal from 1/2 nali, i.e., 100m² area) and garlic yield (30 kg out of 4x4 sq meter area plot) were obtained. | Hansi devi Negi, Adhyaksha (Chairperson) of Mahila Mangal Dal at village Lodh, block Takula, district Almora, “Exploring Livelihood Potential of wild stinging nettle (Uritica dioica)” in Uttarakhand funded by Ministry of Environment, Forest and climate change Government of India under National Mission of Himalayan Studies (NMHS). |  |  |  |
| In the 2020 crop season (March-October), a field experiment was conducted in a randomized block design with three replications to evaluate the efficacy of chemical fungicides, bio-agents and herbal Kunapajal. The experiment comprised of 13 treatments and an untreated control with 5x5 m plot size with nine fungicides. Application of Kunapajal found most effective in promoting plant growth parameters (e.g., plant height, number of tillers and number leaves).Herbal Kunapajala was also found effective in reducing disease severity to 66.21% over the control plots. | Dr Laxmi Rawat, Asst Professor et. Uttarakhand University of Horticulture and Forestry, Bharsar “Usefulness of Herbal Kunapajala in managing stem (rhizome) rot of ginger” |  |  |  |
